# Trends in Body Mass Index, Blood Pressure, and Serum Lipids in Japanese Children: Iwata Population-Based Annual Screening (1993–2008)

**DOI:** 10.2188/jea.JE20090079

**Published:** 2010-05-05

**Authors:** Katsuyasu Kouda, Harunobu Nakamura, Nobuhiro Nishio, Yuki Fujita, Hiroichi Takeuchi, Masayuki Iki

**Affiliations:** 1 Department of Public Health, Kinki University School of Medicine, Osaka-Sayama, Osaka, Japan; 2 Department of Health Promotion and Education, Graduate School of Human Development and Environment, Kobe University, Kobe, Japan; 3 Department of Public Health, Wakayama Medical University, Wakayama, Japan; 4 Hamamatsu University School of Medicine, Hamamatsu, Shizuoka, Japan

**Keywords:** blood pressure, child, cholesterol, epidemiology, risk factors

## Abstract

**Background:**

Current trends in body size, blood pressure, and serum lipids in children are predictors of future disease prevalence. However, there have been no studies of blood pressure and high-density lipoprotein cholesterol (HDL-C) levels in Japanese children.

**Methods:**

We investigated trends in body mass index (BMI), systolic blood pressure (SBP), diastolic blood pressure (DBP), total cholesterol (TC), non-HDL-C, and HDL-C using data from annual screenings in 1993 through 2008. The subjects were 14 872 (98.8% of the target population) fifth-graders enrolled in all public schools in the Original Iwata area in Iwata City, Japan. The same examination protocol was used throughout to ensure the uniformity of quality control and the precision of assessment. Trends in the variables in relation to the calendar year were analyzed by using regression models.

**Results:**

In boys, the 95th percentile of BMI increased by 0.09 kg/m^2^/year. In both sexes, the 5th percentile of BMI decreased by 0.02 to 0.03 kg/m^2^/year. There was a significant negative correlation between SBP and calendar year, and the 95th percentile of SBP decreased by 0.52 mm Hg/year in boys and by 0.40 mm Hg/year in girls. There was also a significant reduction DBP. However, there were no trends in TC, non-HDL-C, or HDL-C.

**Conclusions:**

The increase in obese and underweight children in Original Iwata was consistent with the findings of a nationwide survey. Although high blood pressure and related risk factors were formerly a serious problem in Japan, blood pressure levels have decreased in schoolchildren from Iwata over the past 15 years.

## INTRODUCTION

There is a longitudinal association between obesity as a child and obesity in adulthood.^1^^–^^3^ Being overweight in childhood is also related to risk factors for coronary heart disease (CHD) in adulthood.^4^ Indeed, investigation of a very large cohort of Danes has demonstrated that a higher body mass index (BMI) during childhood is associated with an increased risk of CHD.^5^ Tracking of blood pressure and serum cholesterol concentrations from childhood to adulthood have also been reported.^6^^,^^7^ Furthermore, a cohort study performed in Finland showed that carotid artery intima-media thickness in adults is associated with systolic blood pressure (SBP) and low-density lipoprotein cholesterol (LDL-C) level during childhood.^8^ In addition, a cohort study of a mixed black and white community in Bogalusa, Louisiana in the United States demonstrated that childhood blood pressure is a predictor of arterial stiffness in young adults.^9^ Therefore, recent trends in BMI, blood pressure, and serum lipids in children are likely to be important predictors of subsequent cardiovascular disease trends in adults.

The prevalence of obesity increased in Japanese children from 1960 to 1996 according to the nationwide school health program conducted by the Ministry of Education.^10^ An increase in total cholesterol (TC) in Japanese schoolchildren was also detected from 1960 to 1990 by nationwide surveys.^10^ Likewise, we reported an increase in TC levels in fifth-grade schoolchildren undergoing annual health screening from 1993 to 2001.^11^ However, there have been no reports on secular trends in blood pressure and high-density lipoprotein cholesterol (HDL-C) in Japanese schoolchildren, so changes in blood pressure and HDL-C in this age group are unclear. Therefore, we investigated secular trends in BMI, SBP, diastolic blood pressure (DBP), TC, non-HDL-C (TC minus HDL-C), and HDL-C in fifth-graders, using data from population-based annual screening in Iwata from 1993 through 2008.

## METHODS

### Study population

This study was approved by the Ethical Committee of Kinki University School of Medicine. The city of Iwata is located in Shizuoka prefecture, about 230 km from Tokyo, Japan. On 1 April 2005, the city of Iwata merged with 4 other municipalities, and a new “Iwata City” was created. In the present study, the subjects were fifth-graders who attended elementary schools located in the original Iwata city area (Original Iwata), which covered an area of 64 km^2^ and had a population of 86 700 in 2000. Approximately 5% of the city’s residents work in primary industry, 45% work in secondary industry, and 50% work in tertiary industry.^11^ In the year 2000, the overall Japanese national workforce rates were 5% in primary industry, 30% in secondary industry, and 65% in tertiary industry.^12^

There were 11 public elementary schools and no private elementary schools in the Original Iwata area and all of the children who lived in the area were enrolled in local public schools. All of these schools were controlled by the Iwata Board of Education, which conducted health screening of all fifth-graders in the Original Iwata area every year from 1993 through 2008. There were 15 029 fifth-graders attending the schools in this area, and 14 872 (98.8%) of these children participated in health screening examinations from 1993 through 2008 (Table 1). We analyzed BMI, SBP, DBP, TC, non-HDL-C, and HDL-C levels of these 14 872 children in the present report.

**Table 1.** Characteristics of fifth-grade children participating in annual health examinations

Year	Number	Height (cm)	Weight (kg)	BMI (kg/m^2^)	SBP (mm Hg)	DBP (mm Hg)	TC (mg/dL)	Non-HDL-C (mg/dL)	HDL-C (mg/dL)
Boys									
1993	513	138 ± 6	34 ± 7	17 ± 3	113 ± 11	62 ± 7	168 ± 26	110 ± 24	58 ± 12
1994	569	138 ± 6	33 ± 6	17 ± 2	114 ± 12	62 ± 8	175 ± 25	109 ± 23	66 ± 14
1995	524	138 ± 6	33 ± 6	17 ± 2	114 ± 12	62 ± 10	168 ± 25	99 ± 24	69 ± 15
1996	552	138 ± 6	34 ± 6	18 ± 2	114 ± 11	61 ± 8	172 ± 27	105 ± 24	67 ± 15
1997	506	138 ± 6	33 ± 7	17 ± 3	112 ± 12	59 ± 9	174 ± 26	107 ± 24	66 ± 14
1998	527	139 ± 6	34 ± 7	18 ± 3	110 ± 12	59 ± 8	170 ± 25	106 ± 23	64 ± 14
1999	468	138 ± 6	34 ± 8	18 ± 3	110 ± 12	58 ± 9	173 ± 26	110 ± 25	63 ± 13
2000	440	138 ± 6	33 ± 7	17 ± 3	109 ± 12	58 ± 9	174 ± 26	110 ± 25	64 ± 14
2001	452	138 ± 6	34 ± 7	18 ± 3	107 ± 13	57 ± 9	171 ± 25	108 ± 23	63 ± 13
2002	496	139 ± 6	34 ± 7	18 ± 3	110 ± 12	59 ± 9	177 ± 27	113 ± 25	65 ± 13
2003	415	138 ± 6	34 ± 8	18 ± 3	108 ± 12	58 ± 9	172 ± 25	108 ± 23	64 ± 13
2004	463	139 ± 6	34 ± 7	18 ± 3	109 ± 13	59 ± 9	171 ± 27	107 ± 23	64 ± 13
2005	476	138 ± 6	34 ± 7	18 ± 3	107 ± 12	58 ± 9	171 ± 26	107 ± 24	63 ± 13
2006	417	138 ± 6	33 ± 7	17 ± 3	109 ± 12	59 ± 8	172 ± 27	106 ± 25	66 ± 13
2007	439	138 ± 6	34 ± 8	18 ± 3	107 ± 11	60 ± 8	176 ± 29	109 ± 27	66 ± 12
2008	406	139 ± 6	34 ± 8	18 ± 3	106 ± 11	56 ± 8	170 ± 27	105 ± 24	65 ± 12

Girls									
1993	485	139 ± 6	33 ± 6	17 ± 2	114 ± 11	62 ± 7	166 ± 26	111 ± 24	55 ± 11
1994	567	139 ± 6	33 ± 6	17 ± 2	117 ± 12	64 ± 8	177 ± 27	115 ± 25	62 ± 12
1995	567	139 ± 7	33 ± 6	17 ± 2	115 ± 12	63 ± 9	168 ± 29	104 ± 28	64 ± 14
1996	480	139 ± 7	33 ± 6	17 ± 2	117 ± 11	63 ± 8	173 ± 27	109 ± 25	63 ± 14
1997	537	140 ± 7	34 ± 7	17 ± 2	115 ± 12	62 ± 9	173 ± 26	108 ± 23	65 ± 13
1998	464	140 ± 7	34 ± 7	17 ± 3	114 ± 12	61 ± 8	171 ± 28	109 ± 27	62 ± 14
1999	463	140 ± 6	34 ± 7	17 ± 3	112 ± 12	61 ± 9	174 ± 27	114 ± 24	61 ± 13
2000	401	140 ± 7	34 ± 6	17 ± 2	111 ± 12	59 ± 9	174 ± 24	113 ± 22	61 ± 13
2001	414	139 ± 7	34 ± 7	17 ± 2	110 ± 14	60 ± 10	172 ± 26	111 ± 25	61 ± 12
2002	398	140 ± 7	34 ± 7	17 ± 2	113 ± 12	62 ± 9	176 ± 28	114 ± 25	62 ± 12
2003	399	139 ± 7	33 ± 7	17 ± 2	111 ± 12	60 ± 9	174 ± 26	109 ± 24	64 ± 13
2004	412	140 ± 7	34 ± 7	17 ± 2	109 ± 13	59 ± 9	166 ± 24	106 ± 22	60 ± 11
2005	420	139 ± 6	33 ± 6	17 ± 2	111 ± 12	61 ± 9	170 ± 26	108 ± 24	62 ± 12
2006	391	139 ± 7	33 ± 7	17 ± 2	112 ± 12	61 ± 9	174 ± 28	110 ± 23	64 ± 12
2007	394	140 ± 7	34 ± 7	17 ± 2	108 ± 10	61 ± 8	171 ± 25	107 ± 24	64 ± 11
2008	417	140 ± 7	34 ± 7	17 ± 3	108 ± 11	59 ± 8	171 ± 26	109 ± 23	62 ± 12

BMI, body mass index; SBP, systolic blood pressure; DBP, diastolic blood pressure; TC, total cholesterol; Non-HDL-C, non-high-density lipoprotein cholesterol; HDL-C, high-density lipoprotein cholesterol.

Data are expressed as the mean ± standard deviation.

### Examinations

Health examinations were conducted at each school from April through June every year. The same protocol was used throughout the period from 1993 through 2008 to ensure uniform quality control and precision of the blood tests.

Measurements of height and body weight were made by special teachers (*Yogo* teachers) who have a Japanese national educational license and play a role in health education and health care at schools; all procedures were conducted in accordance with Japanese School Health Law. Height was measured to an accuracy of 0.1 cm and weight to 0.1 kg.^13^ Then BMI (kg/m^2^) was calculated as the weight in kilograms divided by the square of the height in meters.

Resting SBP and DBP were measured by nurses and medical technologists from the *Shizuokaken Yoboigakukyokai* (Shizuoka Prefecture Preventive Medicine Association, Shizuoka, Japan) using an automated oscillometric blood pressure device (BP-103N or BP-103i II, Colin Corporation, Komaki, Japan). The cuff size was selected based on the circumference of the child’s arm. Measurement was done in the seated position with the right arm supported at the level of the heart. If the value obtained was greater than the cutoff point (SBP ≥135 mm Hg or DBP ≥80 mm Hg),^14^ the measurement was repeated. If the second value was still above the cutoff point, a third measurement was obtained. If the third value was also above the cutoff point, the lowest of the 3 values was adopted.

Blood samples were collected by nurses and medical technologists who were also staff of the *Shizuokaken Yoboigakukyokai*, and all testing was conducted at a laboratory of the organization. TC was determined by an enzymatic method (Pureauto S CHO-N, Daiichi Pure Chemical Co., Ltd., Tokyo, Japan) using a Hitachi 7350 automatic analyzer. HDL-C was determined by the direct method (Cholestest N HDL, Daiichi Pure Chemical Co., Ltd.) using the same analyzer. The precision and accuracy of the lipid assays were monitored by internal quality control and by external quality assessment performed by the Japan Medical Association. Coefficients of variation were less than 4%.

### Statistical analysis

Statistical analysis was performed with SPSS Statistics 17.0 for Windows software (SPSS Japan Inc., Tokyo, Japan). The 95th, 90th, 75th, 50th, 25th, 10th, and 5th percentiles of BMI, SBP, DBP, TC, non-HDL-C, and HDL-C were calculated for each year. Regression analysis was performed to evaluate the secular trends in BMI, blood pressure, and serum lipids from 1993 through 2008, with the calendar year of the examination as the independent variable, and the 95th, 50th, and 5th percentile values as the dependent variables. The weighted least-squares method was used to adjust the sample size of each year for regression analysis.

The trend in TC from 2002 through 2008 was also analyzed, because the association between TC and calendar year seemed to be negative from 2002 through 2008, whereas the association between TC and calendar year was previously reported to be positive in boys and girls from 1993 to 2001.^11^ Simple regression analysis was used to evaluate the trend in TC from 2002 through 2008, with the year of the study as the independent variable and TC in each individual as the dependent variable.^11^

## RESULTS

### BMI

**Figure 1.**
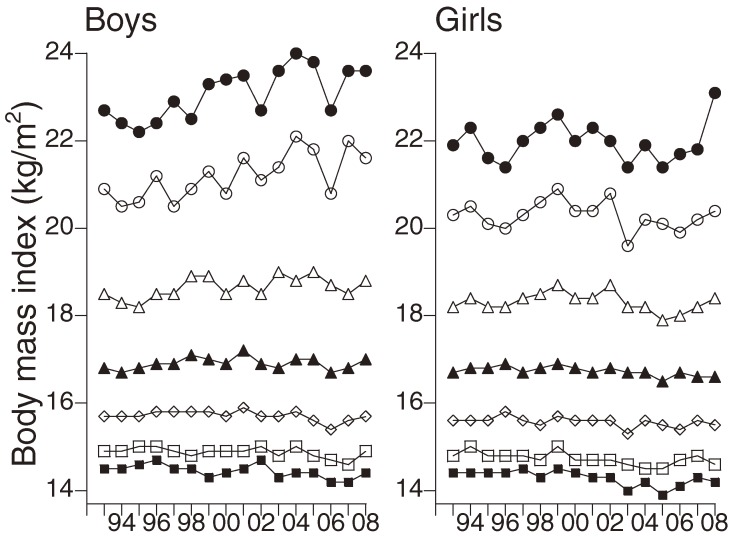
Changes in the percentiles of body mass index. Closed circles, 95th percentile; open circles, 90th percentile; open triangles, 75th percentile; closed triangles, 50th percentile; rhombuses, 25th percentile; open squares, 10th percentile; closed squares, 5th percentile.

In boys, the 95th percentile of BMI was significantly associated with the calendar year over the period from 1993 through 2008. Regression analysis indicated that there was an increase in BMI of 0.09 kg/m^2^/year. In contrast, the 5th percentile of BMI for boys showed a significant reduction of 0.02 kg/m^2^/year during the 1993–2008 period. In girls, both the 50th and 5th percentiles of BMI showed a negative correlation with calendar year during the 1993–2008 period. Regression analysis indicated that there was a decline of 0.03 kg/m^2^/year in the 5th percentile of BMI and a decrease of 0.01 kg/m^2^/year in the 50th percentile (Table 2, Figure 1).

**Table 2.** Secular trends in body mass index, blood pressure, and serum lipids

	Regression coefficient (95% CI)	
	
	Boys (*n* = 7663)	Girls (*n* = 7209)
BMI (kg/m^2^)		
95th percentile	0.087 (0.039 to 0.135)^a^	0.010 (−0.045 to 0.066)
50th percentile	0.006 (−0.010 to 0.023)	−0.012 (−0.023 to −0.001)^a^
5th percentile	−0.017 (−0.030 to −0.004)^a^	−0.025 (−0.039 to −0.011)^a^
SBP (mm Hg)		
95th percentile	−0.524 (−0.815 to −0.234)^a^	−0.401 (−0.610 to −0.192)^a^
50th percentile	−0.484 (−0.713 to −0.256)^a^	−0.506 (−0.710 to −0.301)^a^
5th percentile	−0.565 (−0.780 to −0.350)^a^	−0.507 (−0.790 to −0.223)^a^
DBP (mm Hg)		
95th percentile	−0.141 (−0.328 to 0.047)	−0.049 (−0.196 to 0.097)
50th percentile	−0.326 (−0.489 to −0.163)^a^	−0.211 (−0.406 to −0.016)^a^
5th percentile	−0.375 (−0.624 to −0.126)^a^	−0.365 (−0.606 to −0.125)^a^
TC (mg/dL)		
95th percentile	0.305 (−0.248 to 0.859)	0.030 (−0.601 to 0.662)
50th percentile	0.003 (−0.334 to 0.341)	0.031 (−0.417 to 0.479)
5th percentile	0.090 (−0.247 to 0.427)	−0.070 (−0.441 to 0.302)
Non-HDL-C (mg/dL)		
95th percentile	0.348 (−0.182 to 0.878)	−0.030 (−0.625 to 0.566)
50th percentile	0.004 (−0.356 to 0.365)	−0.159 (−0.524 to 0.206)
5th percentile	0.118 (−0.278 to 0.515)	0.027 (−0.402 to 0.456)
HDL-C (mg/dL)		
95th percentile	−0.118 (−0.595 to 0.359)	0.004 (−0.483 to 0.490)
50th percentile	0.121 (−0.208 to 0.451)	0.196 (−0.089 to 0.480)
5th percentile	0.060 (−0.156 to 0.277)	0.289 (0.047 to 0.532)^a^

Regression analysis was performed to evaluate secular trends.

The independent variable was the year of the study; the dependent variables were the 5th, 50th, and 95th percentiles of each variable.

The weighted least-squares method was used to adjust the sample size for the year.

CI, confidence interval; BMI, body mass index; SBP, systolic blood pressure; DBP, diastolic blood pressure; TC, total cholesterol; Non-HDL-C, non-high-density lipoprotein cholesterol; HDL-C, high-density lipoprotein cholesterol.

^a^*P* < 0.05.

### Blood pressure

**Figure 2.**
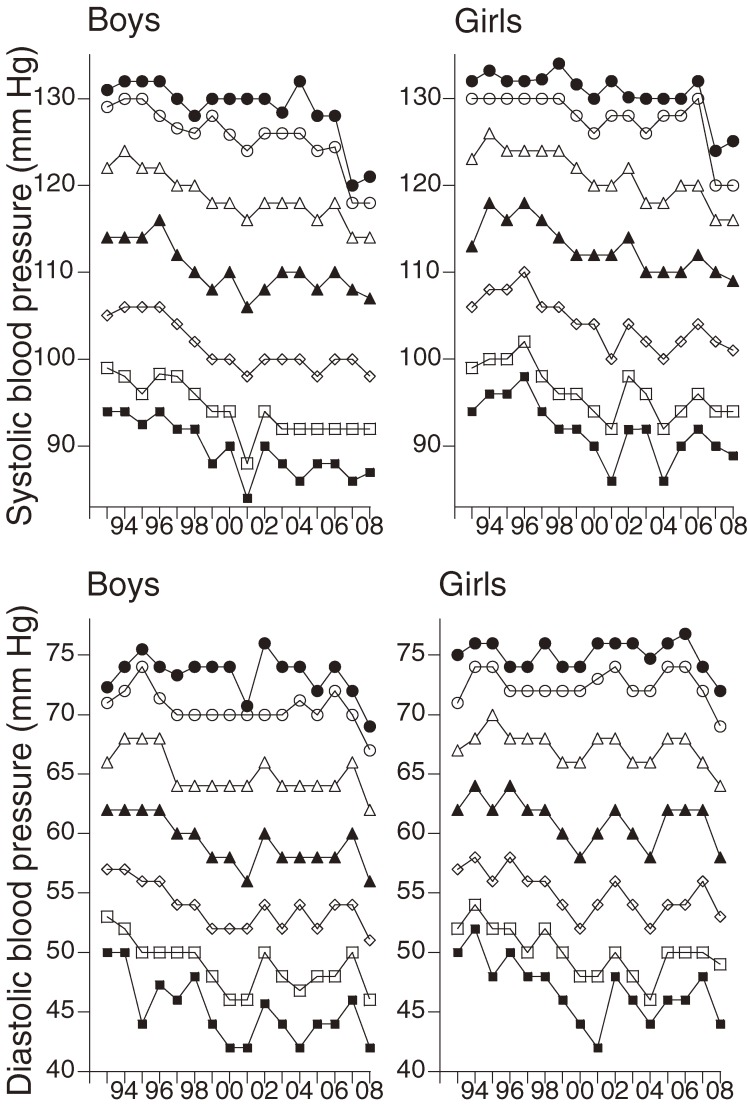
Changes in the percentiles of blood pressure. Closed circles, 95th percentile; open circles, 90th percentile; open triangles, 75th percentile; closed triangles, 50th percentile; rhombuses, 25th percentile; open squares, 10th percentile; closed squares, 5th percentile.

There was a significant negative correlation between the 95th, 50th, and 5th percentiles of SBP and the calendar year from 1993 through 2008 in both sexes. The 95th percentile decreased by 0.52 mm Hg/year in boys and by 0.40 mm Hg/year in girls from 1993 through 2008, while the 50th percentile decreased by 0.48 mm Hg/year in boys and by 0.51 mm Hg/year in girls during the same period. The 5th percentile also markedly decreased in both sexes. Furthermore, there was a significant negative correlation between both the 50th and 5th percentiles of DBP in both sexes and the year from 1993 through 2008 (Table 2, Figure 2).

### Serum lipids

**Figure 3.**
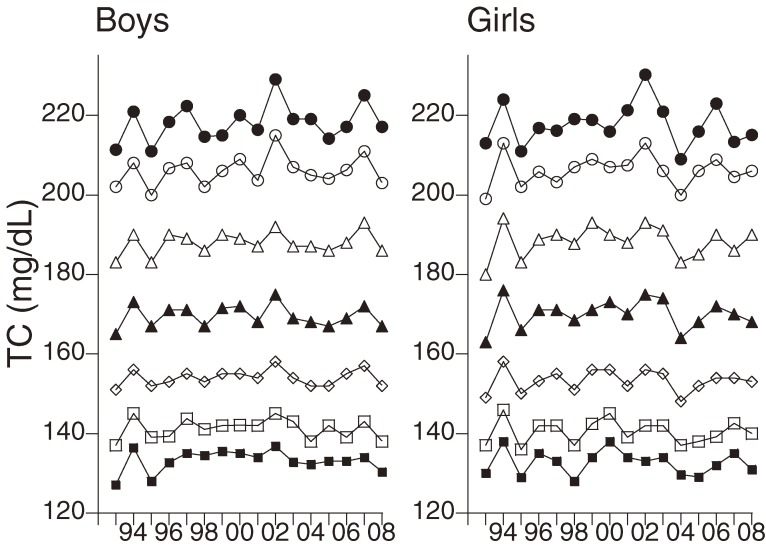
Changes in the percentiles of total cholesterol (TC). Closed circles, 95th percentile; open circles, 90th percentile; open triangles, 75th percentile; closed triangles, 50th percentile; rhombuses, 25th percentile; open squares, 10th percentile; closed squares, 5th percentile.

**Figure 4.**
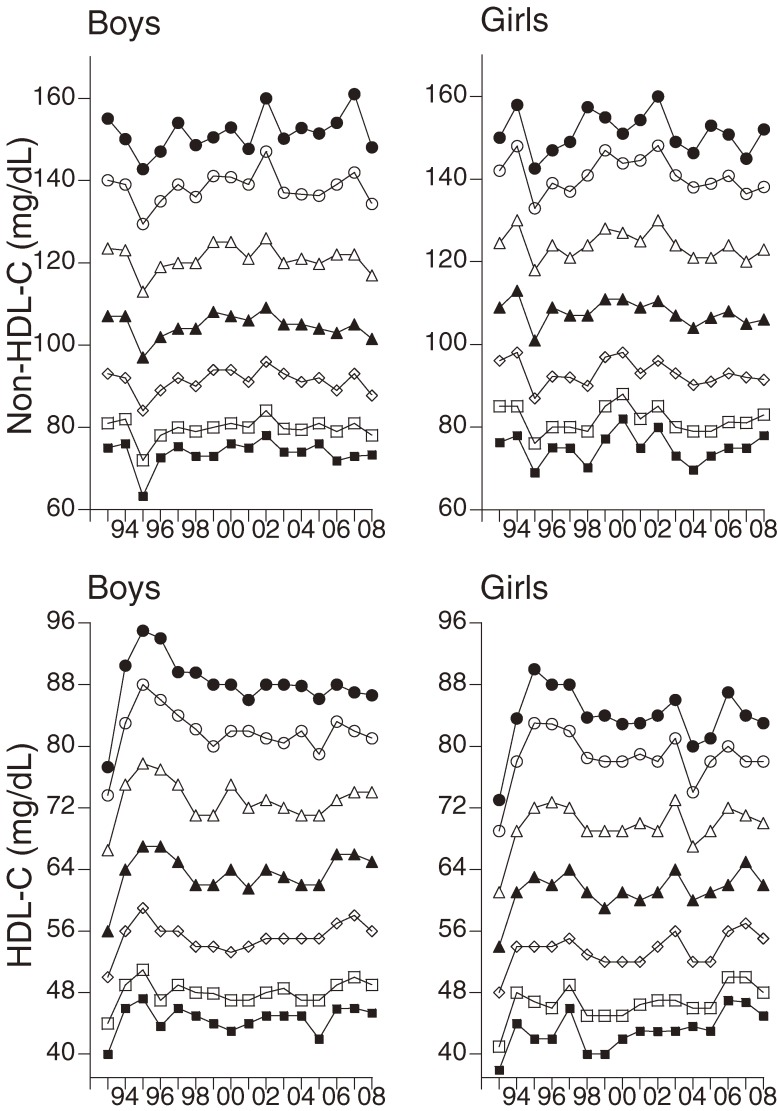
Changes in the percentiles of non-high-density lipoprotein cholesterol (non-HDL-C) and high-density lipoprotein cholesterol (HDL-C). Closed circles, 95th percentile; open circles 90th percentile; open triangles, 75th percentile; closed triangles, 50th percentile; rhombuses, 25th percentile; open squares, 10th percentile; closed squares, 5th percentile.

There were no significant correlations between the percentile values of TC and the calendar year from 1993 through 2008 in either boys or girls (Figure 3). Analysis of trends from 2002 through 2008 showed that TC level tended to decrease. Regression analysis of the relation between TC values and calendar year showed an annual decrease of 0.469 mg/dL (*P* = 0.053) in boys and 0.383 mg/dL (*P* = 0.123) in girls. However, non-HDL-C percentiles were not significantly associated with the year (Figure 4). In girls, the 5th percentile of HDL-C was significantly associated with the calendar year from 1993 through 2008 (Table 2, Figure 4). However, HDL-C values obtained in 1993 seemed to differ from those for 1994 through 2008, and the existence of instrument bias (a common problem during the first year of screening) cannot be excluded. Indeed, there was no significant correlation between the 5th percentile values of HDL-C in girls and the calendar year from 1994 through 2008 (B = 0.069, *P* = 0.764).

## DISCUSSION

This is the first report on secular trends in blood pressure, HDL-C, and non-HDL-C levels in Japanese schoolchildren. Our findings revealed a decrease in the 95th percentile of SBP in both boys and girls during the past 15 years. As a result, there was a decrease in the prevalence of high blood pressure during this period. There was no significant trend in TC, non-HDL-C, or HDL-C from 1993 through 2008.

According to the national school health statistics published by the Japanese Ministry of Education, Culture, Sports, Science and Technology, the mean height of 10-year-old boys was 139 cm in both 1993 and 2008, while that of 10-year-old girls was 140 cm in both years. The mean body weight of boys was 34 kg in both 1993 and 2008, and that of girls was also 34 kg in both years.^15^ The height and weight of Iwata children (Table 1) were similar to the national averages for Japanese children.

The 95th percentile of BMI, which is sometimes used as a cutoff point to identify obesity,^16^ markedly increased over the past 15 years in boys, which indicates that there was an increase in the prevalence of obesity during the period and that obesity remains a serious problem in the Original Iwata community. In contrast, the 5th percentile of BMI (which has been used by the WHO Expert Committee^17^ as a cutoff point to identify excessive thinness in adolescence) decreased in both boys and girls. Thus, underweight also seems to have become more prevalent in both boys and girls from Original Iwata. The nationwide prevalence of obese and underweight Japanese children can be obtained from the school health statistics provided by the Ministry of Education, Culture, Sports, Science, and Technology of Japan.^15^ The prevalence of obesity was 5.9% in 1977, 10.0% in 1993, and 11.3% in 2008 for fifth-grade boys, and 5.8% in 1977, 8.2% in 1993, and 9.4% in 2008 for fifth-grade girls.^15^ Thus, our data from Original Iwata regarding the increase of the 95th percentile in boys were consistent with the national trend in obesity, as determined by nationwide survey, during the same period. The nationwide statistics also showed that the prevalence of underweight among fifth-grade boys was 2.1% in 1993 and 2.4% in 2008, while that for girls was 1.6% in 1993 and 2.4 in 2008.^15^ Thus, data from the Original Iwata area regarding the decrease in the 5th percentile of BMI in boys and girls were also consistent with the secular trend in underweight identified in the nationwide survey during the same period. It has been reported that most Japanese girls overestimate their body weight and want to be thinner.^18^ An association between body image and various lifestyle factors has also been reported.^19^ Therefore, excessive concern about weight among Japanese girls might a reason for the increased prevalence of underweight in Japan.

Childhood hypertension is related to an increase in risk factors in adulthood.^6^^,^^8^^,^^9^ Our regression analyses of data for the present population revealed that both SBP and DBP declined over the period from 1993 through 2008. The 95th percentile of SBP decreased by 0.40 to 0.52 mm Hg every year and the 50th percentile declined by 0.48 to 0.51 mm Hg annually in both boys and girls. The 50th percentile of DBP also decreased by 0.21 to 0.33 mm Hg annually in both boys and girls. The Japanese Ministry of Health and Welfare conducted a national survey of adolescents aged 15 to 19 years and reported that the average SBP of males was 116 mm Hg in 1997 and 114 mm Hg in 2006, while that of females was 110 mm Hg in 1997 and 106 mm Hg in 2006.^20^ Thus, our findings are consistent with the national data for adolescents from 1997 to 2006. This decline in SBP might be partly due to a change in dietary habits, such as a decrease in salt intake. According to the National Nutrition Survey, daily salt intake in Japan was 14.0 g in 1975, 13.0 g in 1980, 12.5 g in 1990, and 10.8 g in 2006.^20^ In addition, the daily salt intakes of Japanese children (age 7–14 years) was 11.4 g in 1996 and 9.7 g in 2006.^20^^,^^21^ Therefore, it can be assumed that salt intake also declined among children from Original Iwata. This suggests that control of blood pressure seems to have been successful in Japan, but careful monitoring of blood pressure is needed to detect more precise changes.

Previous nationwide surveys of Japanese children have shown that serum cholesterol levels have increased from 1960 to 1990.^10^ We previously found a positive correlation between TC levels and calendar year in fifth-grade boys and girls in Original Iwata from 1993 through 2001.^11^ However, in the present study, linear regression analysis revealed no significant association between TC and calendar year from 1993 through 2008. The reason for the lack of a significant trend was the decrease in TC from 2002 through 2008. There was a negative, nonsignificant, association between TC levels and the calendar year from 2002 through 2008. Thus, the TC trend followed a curve in both boys and girls. A decrease in serum TC in adults was identified in the National Nutrition Survey. The average TC level of men aged 20 to 29 years was 183 mg/dL in 2002 and 180 mg/dL in 2006, while the levels for women were 184 mg/dL in 2002 and 181 mg/dL in 2006.^20^ The decline in TC in schoolchildren from Iwata between 2002 and 2008 is consistent with data on adults from the National Nutrition Survey. There was no significant trend in non-HDL-C in the present survey. From these findings, we conclude that the increment of TC from 1960 to 2000 in Japanese children has been partly reversed during the past decade. It has been reported that fat intake has decreased among 10-year-old American children since the mid-1970s,^22^ and that serum cholesterol concentrations have remained relatively stable over the past 2 or 3 decades in American children.^23^ The Japanese National Nutrition Survey showed that the per capital intake of animal fat increased from 1960 to 2000, and then decreased after 2000.^20^ These recent changes in dietary habits might be responsible for the TC levels in Japanese children shown by the present study.

Serum lipid levels were reported for Japanese children from 19 prefectures who underwent a screening and management program for lifestyle-related diseases from 1993 to 1999. In that report, the 50th percentile value for TC was 170 mg/dL in 10-year-old boys and 171 mg/dL in 10-year-old girls, while the 50th percentile value for HDL-C was 62 mg/dL in 10-year-old boys and 61 mg/dL in 10-year-old girls.^24^ Thus, the serum lipid levels of Iwata children (Table 1) were consistent with the reported averages for Japanese children.

This study is the first report on trends in blood pressure, HDL-C, and non-HDL-C in Japanese children. Data were obtained from all 10-year-old children living in a single city by means of annual health screening with a consistent measurement protocol. However, there are some limitations to the present study. The first limitation is that our data were from only 1 area of Japan—the subjects were not randomly selected from across the whole country. The second limitation is that blood pressure measurements were only repeated when the value obtained was greater than the cutoff point. Because of the regression to the mean phenomenon in the children with high blood pressure, the mean value and the 95th percentile might have been underestimated. Therefore, our data on the mean and 95th percentile values of blood pressure should only be used to assess secular trends.

Current trends in risk factors in children are predictors of future disease prevalence. Although hypertension and related diseases were formerly a serious problem in Japan, blood pressure has decreased in schoolchildren from Iwata over the past 15 years. Further investigations are needed to assess the prevalence of obesity, underweight, hypertension, and dyslipidemia in Japanese children.
